# NB-TCM-CHM: Image dataset of the Chinese herbal medicine fruits and its application in classification through deep learning

**DOI:** 10.1016/j.dib.2024.110405

**Published:** 2024-04-10

**Authors:** Dingcheng Tian, Cui Zhou, Yu Wang, Ruyi Zhang, Yudong Yao

**Affiliations:** a College of Medicine and Biological Information Engineering, Northeastern University, Shenyang, 110819, China; b Research Institute for Medical and Biological Engineering, Ningbo University, Ningbo, 315211, China; c Department of Pharmacy, Ningbo Municipal Hospital of Traditional Chinese Medicine, Ningbo, 315010, China; d Department of Electrical and Computer Engineering, Stevens Institute of Technology, Hoboken, New Jersey, USA

**Keywords:** Traditional Chinese medicine, Chinese herbal medicine identification, Deep learning, Image classification

## Abstract

Chinese herbal medicine (CHM) is integral to a traditional Chinese medicine (TCM) system. Accurately identifying Chinese herbal medicine is crucial for quality control and prescription compounding verification. However, with many Chinese herbal medicines and some with similar appearances but different therapeutic effects, achieving precise identification is a challenging task. Traditional manual identification methods have certain limitations, including labor-intensive, inefficient. Deep learning techniques for Chinese herbal medicine identification can enhance accuracy, improve efficiency and lower coats. However, few high-quality Chinese herbal medicine datasets are currently available for deep learning applications. To alleviate this problem, this study constructed a dataset (Dataset 1) containing 3,384 images of 20 common Chinese herbal medicine fruits through web crawling. All images are annotated by TCM experts, making them suitable for training and testing Chinese herbal medicine identification methods. Furthermore, this study establishes another dataset (Dataset 2) of 400 images by taking pictures using smartphones to provide materials for the practical efficacy evaluation of Chinese herbal medicine identification methods. The two datasets form a Ningbo Traditional Chinese Medicine Chinese Herb Medicine (NB-TCM-CHM) Dataset. In Dataset 1 and Dataset 2, each type of Chinese medicine herb is stored in a separate folder, with the folder named after its name. The dataset can be used to develop Chinese herbal medicine identification algorithms based on deep learning and evaluate the performance of Chinese herbal medicine identification methods.

Specifications Table

**Table:** 

Subject	Health and Medical Sciences
Specific subject area	Complementary and alternative medicine, Chinese herbal medicine, Image processing, Image identification, Image classification, Computer vision
Data format	Raw (JPG format)
Type of data	Image
Data collection	First, images of each type of Chinese herbal medicine are searched and downloaded via Google. Then, they are cropped to remove redundant information and unclear images are deleted. Finally, all the images are screened and annotated by professional TCM pharmacists. In addition, we also captured images using smartphones and filtered them, removing any that were unclear.
Data source location	Institution: Ningbo University City: Ningbo Country: China
Data accessibility	Repository name: Mendeley Data Data identification number: 10.17632/2kjmzjyrmd.3 Direct URL to data: https://data.mendeley.com/datasets/2kjmzjyrmd/3

## 1 Value of the Data

• This dataset provides images of 20 common types of Chinese herbal medicine fruits, which can be used as training materials for traditional Chinese medicine practitioners.
• This is the first openly accessible dataset of Chinese herbal medicine fruits. It can provide material for research into the intelligent identification of traditional Chinese herbs.
• This dataset can be used to train deep learning models to recognize different Chinese herbal medicine fruits. This automated recognition ability can be used in pharmacies, warehouses, production lines, and other places for Chinese herbal medicine classification and management, enhancing efficiency and reducing errors.
• This dataset can be used in developing applications for the classification, counting, and quality inspection of Chinese herbal medicine fruits.
• This dataset can be used for performance evaluation of methods in identifying Chinese herbal medicine.

## 2 Background

Chinese herbal medicine is an essential part of TCM, and the correct use of Chinese herbal medicine, including identification and categorization, is crucial to the life safety of patients [1]. In recent years, researchers have gradually used deep learning to recognize Chinese herbal medicines due to significant breakthroughs in image recognition and analysis. Wang et al. [2] constructed a deep convolutional neural network combining the channel and spatial attention modules for the recognition of Chinese herbal medicine. Gang et al. [3] designed a lightweight convolutional neural network for the identification of Chinese herbal medicines and achieved good classification accuracy. Xu et al. [4] used multiple attentional pyramid networks to recognize Chinese herbs. However, although some studies have used deep learning methods to recognize Chinese herbal medicines, the datasets used in these researches are self-built, and the relevant datasets are private. Therefore, comparing the performance differences between these methods on the same dataset is difficult. Meanwhile, the public dataset is an essential factor that promotes the development of related research, so establishing a public dataset of Chinese herbal medicine is significant.

## 3 Data Description

This study has constructed two datasets containing 20 types of Chinese herbal medicine fruits for research on the automatical identification of Chinese herbal medicine. The first dataset (Dataset 1) was obtained through Google search and consisted of 3384 images. The second dataset (Dataset 2) was acquired through taking pictures of Chinese herbal medicine using a smart phone, totaling 400 images.

We obtained images of 20 Chinese herbal medicine fruits through a Google search. After collecting the images, we processed them, deleting unclear images and cropping out redundant information from the images. The 20 classes of images are (1) Psoraleae Fructus, (2) Alpiniae Katsumadai Semen, (3) Toosendan Fructus, (4) Kochiae Fructus, (5) Amomi Fructus Rotundus, (6) Rubi Fructus, (7) Lycii Fructus, (8) Trichosanthis Pericarpoium, (9) Rosae Laevigatae Frucyus, (10) Armeniacae Semen Amarum, (11) Forsythiae Fructus, (12) Chaenomelis Fructus, (13) Amomi Fructus, (14) Crataegi Fructus, (15) Corni Fructus, (16) Persicae Semen, (17) Mume Fructus, (18) Schisandrae Chinensis Fructus, (19) Foeniculi Fructus, (20) Gardeniae Fructus. These image data constitute Dataset 1. In the uploaded dataset, each folder is named after the image name. The dataset contains 3384 images in JPG format, and the image sizes vary.

In addition, images of the above 20 types of Chinese herbal medicine were captured using smartphones, totaling 400 images. Each type of herb has 20 images, forming Dataset 2. After taking all the images, we screened them and removed unclear images. Table 1 shows examples and quantities of each class of data.

**Table 1:** Examples of 20 types of Chinese herb medicine fruits.

Name	Dataset 1		Dataset 2
	Image	Number	
Psoraleae Fructus		177	
Alpiniae Katsumadai Semen		99	
Toosendan Fructus		149	
Kochiae Fructus		280	
Amomi Fructus Rotundus		87	
Rubi Fructus		168	
Lycii Fructus		101	
Trichosanthis Pericarpoium		164	
Rosae Laevigatae Frucyus		146	
Armeniacae Semen Amarum		180	
Forsythiae Fructus		208	
Chaenomelis Fructus		202	
Amomi Fructus		213	
Crataegi Fructus		122	
Corni Fructus		185	
Persicae Semen		178	
Mume Fructus		191	
Schisandrae Chinensis Fructus		179	
Foeniculi Fructus		165	
Gardeniae Fructus		190	

The two datasets form a Ningbo Traditional Chinese Medicine Chinese Herb Medicine (NB-TCM-CHM) Dataset. Within both Dataset 1 and Dataset 2, there are 20 folders each. Each type of Chinese herbal medicine is stored in a separate folder, and the folder is named after the herb's name.

## 4 Experimental Design, Materials and Methods

Accurate identification and utilization of Chinese herbal medicine are crucial for ensuring the efficacy and safety of traditional Chinese medicine treatments [2,5,6]. In recent years, deep learning has achieved outstanding results in image processing. Using deep learning methods can automate the identification of Chinese herbal medicine, thereby improving the accuracy of recognizing Chinese herbal medicine. However, publicly available data are currently scarce. Therefore, we have established a Chinese herbal medicine fruits dataset for training and evaluating deep learning models.

**Fig. 1:**
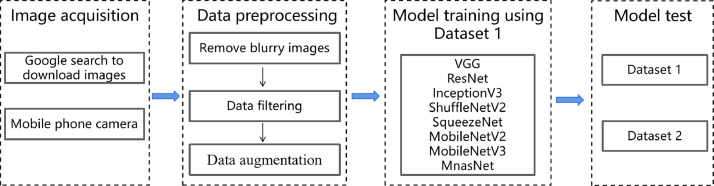
Data processing steps.

In Fig. 1, we show the processing and usage methods of the dataset, which consists of four parts: image acquisition, data preprocessing, model training using Dataset 1, and model test. The image data is obtained through Google search and camera capture. Specifically, firstly, Dataset 1 was obtained through a Google search, and the ``Usage rights'' search tool was used to collect these images during the search. These images are publicly accessible and can be collected. The process was automated using web crawler technology, and after the initial collection, the images were screened by a professional Chinese medicine practitioner to remove the wrong images. Finally, a total of 3384 images of herbal medicines were obtained. A smartphone was used to photograph the herbs when collecting the raw data of Dataset 2. White A4 paper was chosen as the background to minimize interference and enhance the visibility of the subject (Chinese herbal medicines), and the phone was placed on a stand with LED lights for fill light, keeping the light intensity constant during the shooting process. After the shooting, the images were screened, and the unclear images were removed. Finally, a total of 400 images of Chinese herbs were obtained.

After obtaining the images, preprocessing operations are performed on them. Specifically, firstly, the images were screened to remove unclear images. Subsequently, redundant parts of the images were cropped out to remove the interference of background information. Then, the data images obtained through Google search were set as Dataset 1, and the images taken using a smartphone were designated as Dataset 2. Finally, Dataset 1 was divided into training, validation, and test sets. The test set consists of 20 images randomly selected from each category of images. Dataset 2 is all used to test the model performance.

Data enhancement is an essential data preprocessing method. Data augmentation aims to expand the size of the training dataset by making minor changes to existing data [7]. This is an essential step in the deep learning model training process, which can alleviate the problem of insufficient training data, thus reducing the overfitting phenomenon in deep learning model training and improving the neural network's performance. Three data enhancement methods were used in this study: random flipping, random cropping, and rotation. First, the images were uniformly resized to 128 × 128. Then, the images were flipped horizontally on the X and Y axes with a probability of 0.5, respectively. Finally, the image was randomly rotated using the center of the image as the rotation axis. In this experiment, a dynamic data enhancement technique is used. The images are randomly transformed before they are fed into the network for training.

Convolutional Neural Networks (CNN) are among the most common methods in deep learning and have achieved excellent results in image classification. To examine the potential of the dataset in training deep learning models, we used eight classic deep learning models to classify these 20 types of Chinese herbal medicine fruits. These eight deep learning models are VGG16 [8], ResNet18 [9], InceptionV3 [10], ShuffleNetV2 [11], SqueezeNet [12], MobileNetV2 [13], MobileNetV3 [14], and MnasNet [15]. We used four indicators to evaluate the classification performance of the models: AUC, F1-Score, Recall, and Accuracy. Table 2 shows their classification results.

**Table 2:** The classification results of different deep learning models on Dataset 1.

Model	AUC	F1-Score	Recall	Accuracy
VGG16	0.9983	0.9571	0.9572	0.9556
ResNet18	0.9990	0.9657	0.9657	0.9671
InceptionV3	0.9962	0.9373	0.9348	*0.9324*
ShuffleNetV2	0.9987	0.9546	0.9583	0.9517
SqueezeNet	0.9969	0.9355	0.9356	0.9382
MobileNetV2	0.9981	0.9498	0.9508	0.9498
***MobileNetV3***	***0.9973***	***0.9658***	***0.9664***	***0.9710***
MnasNet	0.9995	0.9523	0.9518	0.9536

This study used the PyTorch 2.1 deep learning framework to implement these eight deep learning models. A computer with an Intel i9–10900X CPU Central Processing Unit (CPU) and NVIDIA RTX3090 Graphics Processing Unit (GPU) configuration was used to complete the training and testing of all models. For all classification models, the input image size was set to (128×128×3). During the training process, the initial learning rate was set to 0.0001, the batch size was 64, the epoch was 200, and the optimizer for model training used Adam's algorithm. In addition, Pytorch's ReduceLROnPlateau method was used to adjust the learning rate during training. After completing the training of the models, the model weights that performed best on the validation set were saved for testing. The above settings were kept constant while training all models.

As can be seen from Table 2, among all the models, MobileNetV3 achieved the best classification performance with an AUC of 0.9973, F1 of 0.9658, Recall of 0.9664, and Accuracy of 0.9710. The recognition rate of InceptionV3 was the lowest, with an Accuracy of 0.9324. In addition, we also used Dataset 2 as a test set to evaluate the above-trained deep learning models, and Dataset 1 was used as a training set. Table 3 displays the results of this test. As can be seen from Table 3, MobileNetV2 achieved the best classification performance with an Accuracy of 0.8975. At the same time, all the models achieved satisfactory results, with the accuracy of all models exceeding 80 %, except for SqueezeNet.

**Table 3:** Test results on Dataset 2 using deep learning models trained on Dataset 1.

Model	AUC	F1-Score	Recall	Accuracy
VGG16	0.9941	0.7805	0.8000	0.8000
ResNet18	0.9948	0.8224	0.8225	0.8225
InceptionV3	0.9818	0.8031	0.8250	0.8250
ShuffleNetV2	0.9918	0.8013	0.8125	0.8125
SqueezeNet	0.9887	0.7968	0.7950	*0.7950*
***MobileNetV2***	***0.9978***	***0.8918***	***0.8975***	***0.8975***
MobileNetV3	0.9890	0.8475	0.8500	0.8500
MnasNet	0.9940	0.8030	0.8050	0.8050

There have been some studies focusing on Chinese herbal medicine datasets. For example, Huang et al. [16] constructed a dataset of floral images of Chinese herbal medicines, which can be used to train deep learning models. The dataset contains 12 common floral Chinese herbal medicines, with 1716 original images. Fang et al. [17] built a high-throughput experimental and reference-guided database for Chinese herbal medicines, which can promote the scientific research and drug development of Chinese medicines. Xue et al. [18] constructed a comprehensive Chinese herbal medicines database to connect TCM with modern Western medicine, especially at the molecular level, to understand the mechanism of action of TCM. Although some studies have been conducted to establish databases of Chinese herbal medicines, image datasets of TCM that can be used for deep learning are still scarce. In this study, image data of 20 common fruit-based TCMs were collected, with a total of 3784 images (Dataset 1: 3384, Dataset 2: 400). It was experimentally confirmed that this data could be used for deep learning model training as well as testing. This data is significant for the automatic identification of Chinese herbal medicine.

## Limitations

We have constructed a dataset of Chinese herbal medicine fruits, comprising 20 common types of herbs, suitable for the training and evaluation of deep learning models. However, the dataset still has some limitations. Firstly, the number of images for each type of herb in the dataset is inconsistent, which might pose challenges to the model's training. Secondly, while there is a wide variety of Chinese herbal medicine, we only selected 20 common Chinese herbal medicine fruits. We will continue to establish Chinese herbal medicine datasets in the future, gradually increasing the variety and volume of herbal data.

## Ethics Statement

This study did not conduct experiments involving humans and animals. To collect Google data, we used the “Usage rights” search tool to collect these images. These images are all publicly accessible and can be collected. Furthermore, we applied edits such as cropping to research needs for every image we acquired. After these modifications, the images now differ significantly from their original forms. The authors confirm that the provided data set and presented work strictly meet the ethics requirements for publication in Data in Brief as mentioned in https://www.elsevier.com/authors/journal-authors/policies-and-ethics.

## CRediT authorship contribution statement

**Dingcheng Tian:** Conceptualization, Methodology, Software, Data curation, Writing – original draft. **Cui Zhou:** Methodology, Data curation, Conceptualization, Visualization, Investigation. **Yu Wang:** Software, Validation. **Ruyi Zhang:** Software, Validation. **Yudong Yao:** Methodology, Supervision, Validation, Writing – review & editing.

## Data Availability

NB-TCM-CHM (Original data) (Mendeley Data).
